# Therapeutic misadventure with paracetamol in children

**DOI:** 10.4103/0253-7613.71894

**Published:** 2010-12

**Authors:** So Shivbalan, Malathi Sathiyasekeran, Kuruvilla Thomas

**Affiliations:** Sundaram Medical Foundation, Dr. Rangarajan Memorial Hospital, Shanthi Colony, IV Avenue Annanagar, Chennai 600 040, India

**Keywords:** Acetaminophen, NAC, *N*-acetylcysteine, paracetamol, poisoning, therapeutic misadventure, toxicity

## Abstract

Paracetamol (acetaminophen), though considered a safe, “over the counter” analgesic and antipyretic, can cause liver injury with overdose. Therapeutic misadventure is a unique problem where the existing nomogram used for acute poisoning is not applicable. In this context, early initiation of N-acetylcysteine even before a biochemical evidence of liver injury may be beneficial. A series of 6 children with this type of paracetamol overdose are presented here to increase the awareness and understanding of this problem since no such data is available from India.

## Introduction

Paracetamol (acetaminophen), the globally popular, “over the counter” analgesic and antipyretic, is also an important cause of poisoning. Poisoning, whether suicidal, accidental, or inadvertent, may cause either isolated elevation of transaminases or acute liver failure (ALF).[1] Infants and young children are less susceptible to acute paracetamol toxicity due to the age-associated differences in drug metabolism.[2,3] Therapeutic misadventure is an unintentional overdose of paracetamol that causes unanticipated effects and is sparsely reported from India and may even be considered as nonexistent. Management guidelines for therapeutic misadventure, that is, multiple, supratherapeutic, or multiple recommended doses of paracetamol are not easily accessible.[1,3] We present here a series of children who went through this misadventure.

## Case Reports

All 6 children were hospitalized during April–December 2008 and the decision to assay paracetamol level and administer *N-acetylcysteine* (NAC) was made by the treating physician on clinical grounds. On admission, the blood samples were sent for complete blood count (CBC), liver function test (LFT), prothrombin time (PT), blood glucose, and renal function tests (RFT). Paracetamol assay was performed by modified method of Glynn and Kendal in a standardized quality control certified laboratory using a semi-automated chemistry analyzer (Merck-Microlab 300) and the results were obtained within 1 h of dispatch. The reference range of paracetamol level was 10–20 *μ*g/mL [Table 1].

**Table 1:** Clinical and laboratory profile of paracetamol overdose

*Age/sex*	*Wt (Kg)*	*PCT Dose in mg/kg/day – (Duration of Treatment)*	*CBC*	*LFT*	*P.T*	*PCT level(μg/ml)*	*Last PCT dose*	*NAC#*
54 Days/F	4.3	167 – (2 days)	N	N	N	39	36 h prior	P.O.
18 Mo/F	10.2	367 – (1 day)	N	N	N	120	12 h prior	Initial I.V. loading dose
6 Wks/M	4.9	258 – (1 day)	N	N	N	142	24 h prior	P.O.
3 Yrs/ F	12	90 – (5 days)	N	Ab N	N	32*	48 h prior	P.O.
6 Mo/M	6.3	100 – (Day 1) 120 – (Day 2)	Ab N	Ab N	Ab N	57	36 h prior	Initial I.V. loading dose
7 Mo/ F	7	178 – (4 days)	Ab N	Ab N	Ab N	52*	24 h prior	I.V. only

Wt - weight; PCT - paracetamol; CBC - complete blood count; LFT - liver function test; PT - prothrombin time; Ab N - abnormal; N - normal; P.O. - per oral; I.V. - intravenous; NAC - *N*-acetylcysteine;

* Co-administered with mefenamic acid

# Loading dose of 140 mg/kg followed by 17 doses of 70 mg/kg every 4 h.

### Case 1

A 54-day-old female infant weighing 4.3 kg was brought to emergency with incessant cry. Her mother believed that paracetamol was safer for high temperature and had given 5 mL (120 mg) of paracetamol every 4 h for 2 days with the last dose being 36 h before presentation. The baby was restless but afebrile and the systems were clinically normal. The calculated total dose of paracetamol administered was 170 mg/kg/day and the serum level was 39 *μ*g/mL (normal range 10–20 *μ*g/mL). CBC, LFT, and PT were normal. She was prescribed oral NAC, but the medications were not administered and 48 h later the paracetamol level was still high at 29 *μ*g/mL, although her liver biochemistry was normal. She was hospitalized and the recommended dose of oral NAC was initiated at 150 mg/kg followed by 17 doses of 75 mg/kg every 4 h. She tolerated NAC and her paracetamol level was 5 *μ*g/mL at the time of discharge.

### Case 2

An 18-month-old female infant with a history of previous febrile seizure presented with lethargy and coffee ground vomiting following intake of paracetamol. Her mother, unaware of the higher concentration in the liquid preparation, gave 6 doses of 5 mL (125 mg/mL) paracetamol every 4 h (a total of 367 mg/kg/day) with the last dose being 12 h before presentation raising the paracetamol level to 120 *μ*g/mL. The infant was lethargic and anicteric. Intravenous (I.V.) fluids with ranitidine were administered. The recommended dose of oral NAC was initiated though her CBC, LFT, PT, and renal parameters were normal. She responded well and the paracetamol level normalized in 48 h.

### Case 3

A 6-week-old male infant (weight 4.88 kg) was inadvertently given paracetamol, one dose of 0.6 mL (60 mg) and 2 doses of 6 mL each over 24 h postvaccination by his mother who was ignorant of the correct dosage. The calculated dose of paracetamol given was 258 mg/kg/day and the serum level was 142 μg/mL. His CBC, LFT, PT, and renal parameters were normal. The child was stable and he was given oral NAC. On discharge, the paracetamol level was <10 μg/mL.

### Case 4

A 3-year-old girl presented with vomiting, decreased food intake for 2 days, and wheeze associated with viral infection for 1 week. She had fever for the initial 5 days and was afebrile for 2 days. She was given paracetamol dose of 180 mg (15 mg/kg) every 4 h for initial 3 days, followed by combination with mefenamic acid 25 mg/kg/day for the next 2 days (last dose was 48 h prior). On examination, she had bilateral extensive wheeze. Investigations revealed elevated serum glutamic oxaloacetic transaminase (SGOT) (326 U/L) and serum glutamic pyruvic transaminase (SGPT) (1026 U/L) and normal PT. The paracetamol level was 32 μg/mL and viral markers were negative. NAC was started, paracetamol level decreased to <10 μg/mL at the end of 17 doses, the SGOT was 96 U/L and SGPT was 236 U/L at discharge, which normalized in the next 10 days.

### Case 5

A 6-month-old male infant (weight 6.3 kg) with a past history of febrile seizure presented with 2 days of fever and 1 day of decreased activity and blood-stained vomitus. His mother had meticulously given 5 doses of paracetamol 125 mg (5 mL) on day 1, that is, 100 mg/kg/day and on day 2 62.5 mg (2.5 mL) every 2 h, that is, 120 mg/kg/day. Total bilirubin was 1.1 mg/dL, SGOT 1774 U/L, SGPT 529 U/L, and PT 15.2 s (control 13.5). Paracetamol level was 57 μg/mL (last dose 36 h prior). A diagnosis of dengue fever with paracetamol overdose was made based on clinical features, low platelet count, and positive dengue serology. The child was given the full course of NAC. He recovered and was discharged with normal laboratory parameters.

### Case 6

A 7-month-old girl was referred with a history of high-grade fever for 4 days, lethargy, poor feeding for 2 days, hematochezia, and ooze from I.V. puncture sites. The child was drowsy on examination with poor hemodynamic status, hepatomegaly, and free fluid in the abdomen. She was admitted in the ICU and given supportive therapy. Hemoglobin was 9.3 gm%, PCV 32%, normal white blood cell count, platelet 8000/mm^3^, serum creatinine 1.7 mg/dL, BUN 69 mg/dL, total bilirubin 0.8 mg/dL, SGOT 2102 U/L, SGPT 569 U/L, total protein 3.4 gm/dL, serum albumin 2.3 gm/dL, and PT 23.3 s (control 13.5). Her paracetamol level was 52 μg/mL (last dose was 24 h prior) and Elisa dengue IgM was positive. During illness she had received 5 doses of 250 mg tablets of paracetamol, that is, 178 mg/kg/day (weight 7 kg) along with 3 doses of 100 mg of mefenamic acid per day (ie, 43 mg/kg/day). She was given an I.V. loading dose of NAC. However, within 24 h, her general condition deteriorated and she succumbed to multiorgan failure.

## Discussion

In India, paracetamol toxicity is considered uncommon and is rarely reported in children. In developed countries, paracetamol-induced ALF is an important indication for liver transplantation.[4] Poisoning in children is usually accidental and therapeutic misadventure due to inappropriate dosing is well documented in infants and preschool children.[5,6] In our series, the parents had inadvertently administered the medication and 4 out of 6 (66%) patients were infants. The recommended dose for oral or rectal paracetamol in symptomatic fever (temperature> 38.5°C) is 15 mg/kg every 6 h (≤60 mg/kg/day),[7] whereas the recommendation for analgesia is 15 mg/kg every 4–6 hourly, up to a maximum of 60–90 mg/kg/day for oral dose and the rectal dose being 20 mg/kg/dose every 6 hourly, up to a maximum of 90 mg/kg/day.[7] A sustained administration of supratherapeutic doses of paracetamol (>90 mg/kg/day) to a sick child younger than 2 years for more than 1 day has been identified as a significant risk for hepatotoxicity, whereas in acute ingestion of paracetamol a higher dose of 150 mg/kg is associated with toxicity.[8] In the presence of risk factors, such as underlying febrile illness, prolonged starvation, repeated vomiting, diarrhea, poor fluid intake for more than 24 h, dehydration, age less than 2 years, hepatic impairment, obesity, multiple dosing, and combination with other antipyretic agents, such as ibuprofen, a much lower dose may suffice to cause hepatotoxicity.[7] In this series, all the children had more than one risk factor and 5 of 6 cases had received >90 mg/kg/day of paracetamol. Thus, inadvertent usage of even recommended doses of multiple antipyretics during febrile illness may be hepatotoxic. A recent Australasian guideline has stated that a dose of ≥200 mg/kg/day for the preceding 24 h or ≥150 mg/kg/day for preceding 48 h or ≥100 mg/kg/day for preceding 72 h, in children 0–6 years exposed to multiple, supratherapeutic doses, or multiple recommended doses[9] of paracetamol is associated with hepatic injury. Hence it is recommended that a caution be exercised when >90 mg/kg/day of paracetamol is administered for more than a day to a “sick” child (vomiting, diarrhea, poor oral intake) younger than 2 years of age.[7,10]

Paracetamol is rapidly absorbed from the small intestine and peak serum concentrations are achieved within 1–2 h for tablets and 30 min for liquid preparations. The elimination half-life of paracetamol is 1–3.5 h and the peak serum concentration after therapeutic doses usually does not exceed 130 *μ*mol/L (20 mg/L or 20 *μ*g/mL). This safe cut-off level along with normal aminotransferase may exclude the risk of subsequent hepatotoxicity. However, detection of paracetamol 24 h after the last dose or the estimated elimination half-life >4 h suggests overdosage.[1] In 5 of 6 cases, the levels were well above normal range even 24 h after ingestion of the last dose.

The liver metabolizes about 90% of paracetamol to inactive sulfate and glucuronide conjugates that are excreted in urine. When these pathways are saturated, cytochrome-P450 becomes quantitatively important and results in the formation of highly reactive intermediary compound *N*-acetyl-p-benzoquinone-imine (NAPQI), which is immediately bound by intracellular glutathione and eliminated in urine. Damage to the hepatocytes may be due to the accumulation of NAPQI as well as the depletion of glutathione.[11]

The patients may be asymptomatic for the first 4–6 h following paracetamol poisoning or may have mild symptoms, such as nausea or vomiting. However, with the formation of NAPQI and depletion of hepatic glutathione stores to a critical level, hepatotoxicity ensues. Almost all will develop elevations of the aspartate aminotransferase and alanine transaminase (ALT) within 24–36 h.[12] In general, maximal hepatotoxicity occurs at 72–96 h.[13] A normal LFT therefore does not exclude the possibility of drug overdosage as this may occur over time with depletion of hepatic glutathione stores. This may be the explanation in cases 1, 2, and 3, where despite significant time lag high serum levels of paracetamol were documented but LFT was normal. Deaths usually occur within the first week either due to acute liver or renal failure[11] but those who recover do not progress to chronicity.[14] In this series, one child with dengue illness succumbed to ALF possibly due to viral illness and paracetamol overdose.

**Figure 1:**
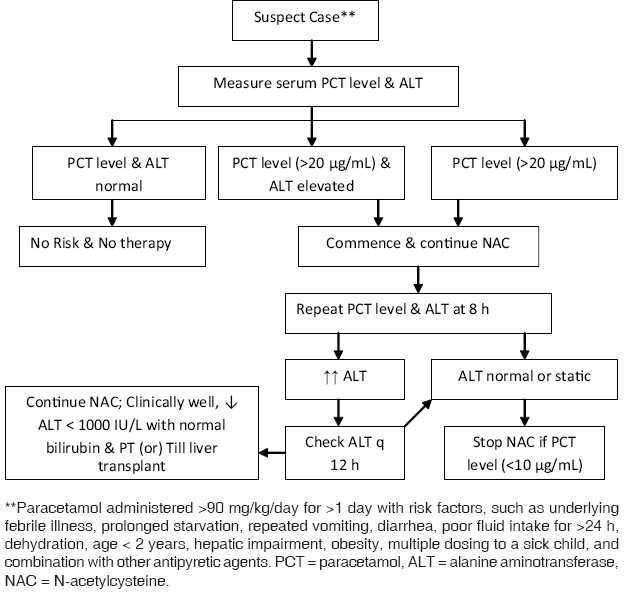
Management algorithm for therapeutic misadventure with paracetamol

NAC is the antidote of choice for paracetamol-induced hepatotoxicity. The therapeutic efficacy of NAC is best when administered within 8 h of acute paracetamol ingestion. The issue is different with repeated supratherapeutic doses or multiple recommended doses of the drug where the existing nomogram may not be applicable. In a suspect case of paracetamol toxicity due to “therapeutic misadventure,” that is, paracetamol administered >90 mg/kg/day for more than 1 day to a “sick” child with risk factors both paracetamol level and liver enzymes should be checked and the child managed as per the algorithm [Figure 1]: (a) NAC is not necessary if both paracetamol (<20 *μ*g/mL) and transaminases are normal. However if there is a delay in obtaining the values NAC can be initiated and discontinued if results are normal. (b) NAC is recommended if both paracetamol (>20 *μ*g/mL) and ALT levels are elevated (>2 times upper limit of normal). (c) NAC is commenced and continued if paracetamol level (>20 *μ*g/mL) is increased and transaminases are normal as there may be delay in the rise of transaminases. The levels are tested every 8 h and NAC is continued till the drug level is undetectable (<10 *μ*g/mL).

NAC acts directly or through its synthesis to cysteine and glutathione.[11] Oral NAC is as efficacious as parenteral therapy and is well tolerated, simple, and cheap with most of the drug delivered to the liver, its site of action. The most common side effects are nausea and vomiting attributed to the rotten egg odor. Oral NAC is given in a loading dose of 140 mg/kg followed by 70 mg/kg every 4 h for 72 h (17 doses). I.V. NAC is indicated when there is risk of aspiration, persistent vomiting, or ALF. It is continued till the drug levels are undetectable (<10 *μ*g/mL) and the patient is clinically well with normal liver function tests. NAC may act as a precursor for glutathione synthesis or it may form complexes with the toxic metabolites of paracetamol. It reverses tissue hypoxia by enhancing tissue oxygen delivery and consumption and may lower the incidence of multiorgan failure in ALF. In ALF, it should be continued till the serum liver transaminases decrease to less than 1000 IU/L, bilirubin and coagulation studies are normal, and the patient is clinically well or until the patient receives a liver transplant.[13] The indications for liver transplantation in paracetamol-related ALF are arterial pH < 7.3, PT > 100 s (international normalized ratio > 4), serum creatinine > 3.5 mg/dL, or rapid progression to hepatic encephalopathy (≥ grade III).[3,4]

Paracetamol is still a safe antipyretic but has the potential for hepatoxicity not only with excessive doses but even with repeated therapeutic doses. The predictors of hepatotoxicity should be recognized and therapeutic misadventure with paracetamol considered an etiology in ALF. A careful history will help in establishing the diagnosis and initiating proper therapy. NAC should be initiated at the earliest since results have been better when administered before severe liver injury. An easy availability over the counter, several preparations with varying drug doses, overzealous usage to suppress fever and its inadvertent use with the notion of being a safe drug contribute to therapeutic misadventure. The recommendations for reducing the risk would be to educate the caregivers about the potential for toxicity. The dosing guidelines based on age and weight should be reviewed by the physician during each visit. A single standard concentration paracetamol product for pediatric age group will also help reduce this malady.
